# Safety and Efficacy of Small Bowel Examination by Capsule Endoscopy for Patients before Liver Transplantation

**DOI:** 10.1155/2017/8193821

**Published:** 2017-01-11

**Authors:** Kawano Seiji, Takaki Akinobu, Iwamuro Masaya, Yasunaka Tetsuya, Kono Yoshiyasu, Miura Kou, Inokuchi Toshihiro, Kawahara Yoshiro, Umeda Yuzo, Yagi Takahito, Okada Hiroyuki

**Affiliations:** ^1^Department of Gastroenterology and Hepatology, Okayama University Graduate School of Medicine, Dentistry, and Pharmaceutical Sciences, Okayama, Japan; ^2^Department of Endoscopy, Okayama University Hospital, Okayama, Japan; ^3^Department of Hepato-Biliary-Pancreatic Surgery, Okayama University Hospital, Okayama, Japan

## Abstract

*Background and Aims*. Gastrointestinal surveillance is a requirement prior to liver transplantation (LT), but small intestine examination is not generally undertaken. The aim of the present study was to evaluate the safety and efficacy of capsule endoscopy (CE) for patients with end-stage liver disease.* Methods*. 31 patients who needed LT were enrolled, and 139 patients who underwent CE over the same period of time acted as controls.* Results*. Frequency of successful achievement of evaluation of the full length of the small bowel, the mean gastric transit time, and the mean small bowel transit time were not significantly different between the two groups. Abnormalities in the small bowel were found in 26 patients. Comparative analysis revealed that history of EV rupture, history of EV treatment, red color sign of EV, and presence of PHG or HCC were significantly associated with patients with >2 two such findings (high score group).* Conclusions*. Small bowel examination by CE in patients before liver transplantation could be performed safely and is justified by the high rate of abnormal lesions detected particularly in patients with history of EV therapy or bleeding, red color sign, and presence of PHG or HCC. This study was registered in the UMIN Clinical Trial Registry (UMIN 000008672).

## 1. Introduction

Liver transplantation (LT) is now routinely performed for various end-stage liver diseases. With improved early-term management, 10-year survival rates of patients transplanted for several different indications exceed 70% [1]. Recently, survival of recipients transplanted for hepatocellular carcinoma (HCC) was reported to be approximately 90%, 85%, and 80% at 1, 3, and 5 years, respectively [2]. LT is also widely used to treat patients with end-stage liver cirrhosis (LC) of various etiologies. Prior to performing LT, endoscopic examinations are necessary to evaluate the state of the gastrointestinal tract. One reason for this is that screening for cancer lesions in the extrahepatic area is absolutely required before surgery. We previously reported the usefulness of colonoscopy prior to LT in patients who can tolerate the procedure [3]. Another reason for pretransplant GI examination is to monitor the possible occurrence of mucosal changes with portal hypertension (PH) in the stomach and colon, as described earlier [4, 5]. In addition to the stomach and colon, the small bowel should also be examined early in the course of the diagnostic workup. De Palma et al. recommended performing CE as a routine examination in Child–Pugh class C patients [6] because portal hypertensive enteropathy (PHE) was frequently found by CE in cirrhotic patients but not in controls (67.5% versus 0, *P* < 0.001). Moreover, these investigators found that esophageal varices ≥ grade 2, portal gastropathy, portal colonopathy, and Child–Pugh class C cirrhosis were all significantly associated with PHE.

Despite the frequency of small intestinal lesions in cirrhotic patients, to the best of our knowledge, there have been no studies evaluating the clinical significance of small bowel CE examination in patients before LT. The aim of the present study was to determine the safety of CE and to determine the prevalence of small bowel lesions in these patients.

## 2. Materials and Methods

### 2.1. Study Design

This study was a nonrandomized, controlled, prospective cohort study. Exclusion criteria were (1) patient's age < 18 years, (2) postponement of LT, (3) emergent LT case, (4) difficulty in performing CE because of severe hepatic encephalopathy or risk of retention, and (5) absence of patient's informed consent for CE. This prospective study was approved by the Ethics Committee of our hospital. Written informed consent was obtained from all patients before CE. This study was registered in the UMIN Clinical Trial Registry (UMIN 000008672). We affirm that all authors had access to the study data and reviewed and approved the final manuscript.

### 2.2. Patients

From April 2012 through March 2015, a total of 38 consecutive patients who needed LT and were treated at Okayama University Hospital were enrolled. The control group consisted of 139 patients who underwent CE during the same period as this study at the same hospital because of obscure gastrointestinal bleeding (*N* = 73), abdominal pain or discomfort (*N* = 25), diarrhea (*N* = 18), anemia (*N* = 13), or other symptoms (*N* = 12, data not shown). All control patients had normal liver biochemistry, viral markers, prothrombin time, and renal function test results. Moreover, they had no history of GI tract surgery, and no gastrointestinal lesions were found by abdominal ultrasonography, upper GI endoscopy, and colonoscopy.

### 2.3. Endoscopic Examination

All patients underwent computed tomography (CT) and abdominal ultrasonography (US) to rule out bowel obstruction as well as to confirm the presence or absence of hepatocellular carcinoma (HCC), portal vein tumor thrombus, and ascites. Upper GI endoscopy and colonoscopy were performed independently, and the presence or absence of esophageal varices (EV), gastric varices (GV), portal hypertensive gastropathy (PHG), and portal hypertensive colonopathy (PHC) was determined. CE was later performed with a video capsule endoscopy device (PillCam SB2; Given Imaging Ltd., Yokneam, Israel). The capsule was swallowed with a solution of dimethicone after an overnight fast, without any other preparation. Two and four hours after swallowing the capsule, patients were allowed to drink clear liquids and to eat a light meal, respectively; after eight hours, the sensor array and recording device were removed. Images were analyzed with Rapid Reader 6 software on a RAPID 6.5 workstation (software and workstation from Given Imaging Ltd.). Two experienced endoscopists made diagnoses after reaching an agreement with each other.

### 2.4. Study Measurements

First, to elucidate the suitability and safety of CE in the study group, the rates of successful achievement of evaluation of the full length of the small bowel, mean gastric and small bowel transit times, and incidence of adverse events were recorded and compared with the control group. Second, the number, type, and location of small bowel lesions found by CE were determined in each patient. The entire small bowel was divided into three regions based on the transit time: the proximal, middle, and distal small bowel. Clinical factors and their association with small bowel lesions were examined; these included sex, age, liver function (Child–Pugh score), etiology of the cirrhosis (viral/nonviral), laboratory test results (hemoglobin, serum ferritin, serum iron, total bilirubin, aspartate transaminase, alanine transaminase, albumin, prothrombin time, and platelet count), model for end-stage liver disease (MELD) score, and presence or absence of EV, GV, PHG, PHC, hepatocellular carcinoma (HCC), portal vein tumor thrombus, or ascites.

### 2.5. Capsule Endoscopy Findings and Scoring System

In this study, we used a modified scoring system with CE, as originally proposed by Abdelaal et al. [7], helpful in grading PHE severity. PHE is divided into the following four types: red spots, angioectagias, varices, and inflammatory-like lesions. Here, we divided inflammatory-like lesions into erosions and villous edemas. Moreover, the presence of lesions with active bleeding was also evaluated. Finally, in the present study, we used a six-point scoring system based on the presence of six different lesions (i.e., red spots, angioectagias, varices, erosions, villous edemas, and active bleeding).

### 2.6. Statistical Analysis

Continuous data were compared using the unpaired Student* t*-test or the Mann–Whitney test. Assessment scores were regarded as ordinal scale scores and analyzed by the Mann–Whitney test. Categorical variables were tested using the corrected chi-squared test. Multivariate analysis was performed using multiple backward stepwise logistic regressions. JMP version 8 (SAS Institute, Cary, NC, USA) was used for all statistical analyses. *P* values <0.05 were considered statistically significant.

## 3. Results

### 3.1. Subject Enrollment and Baseline Characteristics

**Figure 1:**
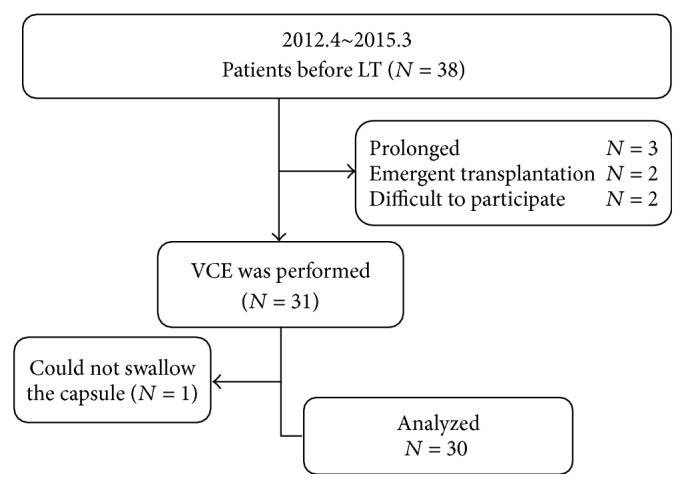
Flow chart of the study.

The study's participant flow plan is shown in Figure 1. Seven patients were excluded because of postponed LT (*N* = 3), refusal to participate in the study (*N* = 2), and emergent LT for fulminant hepatitis (*N* = 2). Consequently, 31 patients were enrolled and CE was performed. One patient was unable to swallow the capsule and canceled the procedure. Then, finally, 30 cases could be analyzed.

The patients' baseline characteristics are shown in Table 1. All patients were categorized as class C according to the Child–Pugh classification system, and their MELD score was high (from 11 to 28 median: 17.0). Endoscopic findings by upper GI and colonoscopy revealed EV (*N* = 22, 73%), GV (*N* = 5, 17%), PHG (*N* = 9, 31%), and PHC (*N* = 9, 31%). Furthermore, 13 patients had a history of endoscopic treatment for EV. In particular, 8 of them required emergent endoscopic treatment for EV owing to bleeding.

### 3.2. Safety of CE for Patients Who Needed LT

Complete evaluation of the small bowel was accomplished in 26 of the 30 patients (87%). The mean gastric transit time was 34.2 ± 10.9 min, and the mean small bowel transit time was 320.5 ± 24.2 min. These results were not significantly different in the study and control groups. One patient only in the study group was unable to swallow the capsule and canceled the procedure, but no other adverse events occurred in either group (Table 2).

### 3.3. Endoscopic Findings

**Figure 2:**
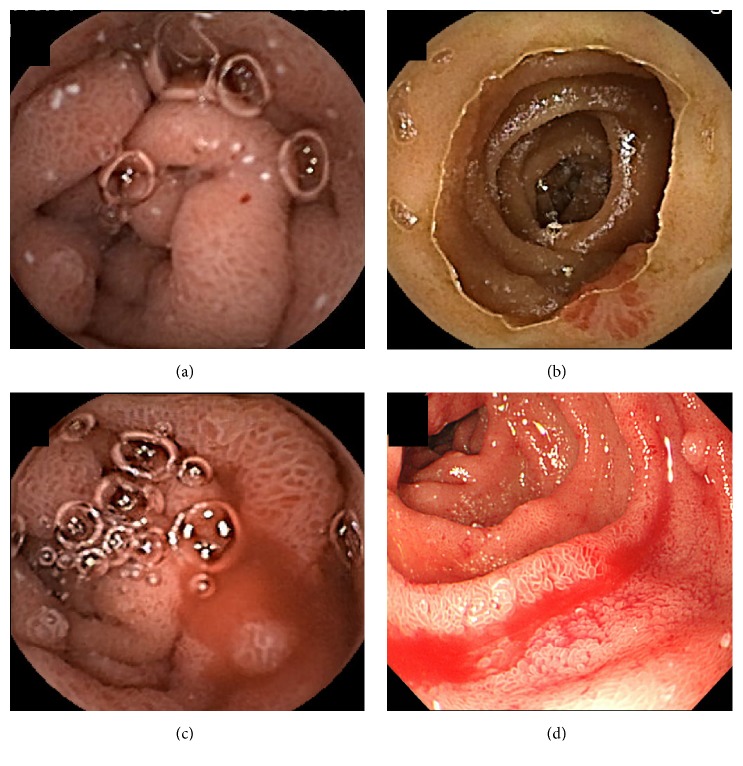
A 53-year-old man had esophageal varices with red color sign and a history of variceal therapy. Villous edema and red spots (a), angioectagia (b), and active bleeding (c) were detected by CE. Active bleeding was confirmed by intestinal endoscopy (d).

Small bowel lesions detected by CE are shown in Table 3. Abnormalities in the small bowel were found in 26 patients (87%), presenting as villous edema (*n* = 20), red spots (*N* = 17), angioectasia (*N* = 8), erosion (*N* = 5), and varices (*N* = 2). We evaluated in this study that red spots (as shown in Figure 2(a)) were equivalent to type 1A by Yano-Yamamoto classification [8], and angioectagias (as shown in Figure 2(b)) were equivalent to type 1B. Active bleeding from jejunal edematous mucosa with red spots was detected in one patient and treated by emergent endoscopy. The patient had EV with red color sign and PHG by EGD. He had a history of EV treatment (Figure 2).

### 3.4. Scoring of CE Findings

**Figure 3:**
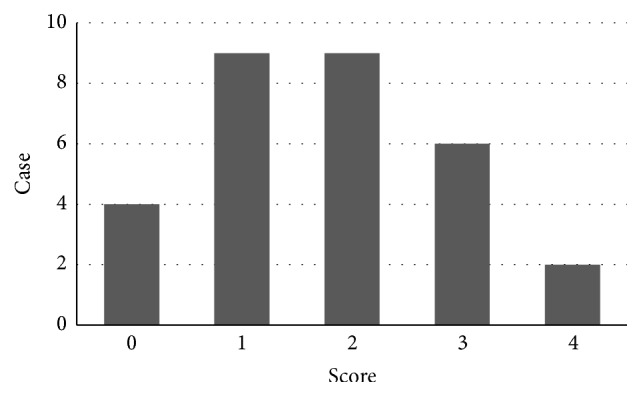
Scoring of CE findings was performed. Each finding (villous edema, red spot, angioectagia, erosion, varices, and active bleeding) was scored 1 point and full score by 6 points.

Scoring of CE findings is shown in Figure 3. Patients in the study group were scored from 0 to 4, with a median score of 2. According to each patient's score, we stratified the subjects into two groups, defining those with a score of 0 or 1 as the low score group and those with a score ≧2 as the high score group. This revealed that history of EV rupture, history of EV treatment, red color sign of EV, and presence of PHG and HCC were all significantly associated with the high score group (Table 4). On the other hand, there were no significant differences between the high and low score groups in clinical laboratory data, such as the Child–Pugh and MELD scores.

## 4. Discussion

Several reports have been published on CE in patients with liver cirrhosis, but in most cases it was performed for compensated-type disease, namely, class A and B diseases, according to the Child–Pugh classification [7, 9–12]. In a paper reviewing previous studies, Mekaroonkamol et al. reported that advanced cirrhosis (Child–Pugh class C) was consistently associated with PHE [13]. However, the number of patients with class C in each of the studies analyzed in that review was small. In contrast, in the present study, all enrolled patients had severe liver dysfunction and needed LT; hence they were all class C. Furthermore, to the best of our knowledge, this is the first study performing CE for patients before LT.

First, we found that successful complete total small bowel examination rate, mean gastric transit time, and mean small bowel transit time were not significantly different between the study and control groups. Liao et al. have reviewed large numbers of cases of CE procedures, concluding that completion rates were 84.8% and 81.3% in prospective studies and retrospective studies, respectively [14]. We therefore consider that our results (86% in the LT group and 92% in control group) are comparable. On the other hand, one patient could not swallow capsule for psychosomatic reasons, but no other adverse events including retention occurred in our series. One objective of this study was to evaluate the safety of CE for patients with end-stage liver disease. From this experience, we do conclude that CE for patients with severe liver dysfunction before LT is perfectly safe.

The main objective of this study was to investigate the feasibility of small bowel examination using CE and to evaluate associations of intestinal lesions with clinical factors. We detected a high rate of abnormal lesions in this study, most of them compatible with the clinical characteristics of PHE that have been previously reported [4–8]. Of these, small intestinal edema was the most common (69%). Takahashi et al. reported that small intestinal edema had the strongest correlation with hepatic venous pressure gradients [15]. Almost all the patients enrolled in the present study had severe liver dysfunction, as they were scheduled for LT. Therefore, almost all of them were considered to have high portal venous pressure.

When we divided all of the patients into two groups using CE scores (low and high score groups), we found that history of EV therapy or bleeding, red color sign, and PHG were more often present in the high score group (Table 4). El-Khayat et al. reported that PHE increased significantly from 6.6% before to 46.7% after variceal obliteration [16]. We also consider from this study that PHE may be worsened after endoscopic therapy of EV. Therefore, we demonstrate that CE should be performed and checked PHE when patients before LT had a history of EV therapy. Furthermore, in the present study, the presence of HCC was more common in the high score group. Faitot et al. reported that portal hypertension was to be regarded as a major risk factor for dropping out of the waiting list and needed to be taken into consideration when managing patients with HCC who were waiting for LT [17]. We especially consider that CE should be performed and PHE should be checked in HCC patients awaiting LT.

We had no case in this study that had to postpone LT due to the CE findings. However, one patient did have active bleeding from PHE of the jejunum (Figure 2). Therefore, we consider that one does need to take account of PHE in all patients before LT. If anemia improved constantly and LT could not be performed within the near future, we consider treatment for small bowel abnormalities in PHE patients.

Several earlier studies documented the feasibility of colonoscopy for screening colorectal tumors before LT. This is a concern because immunosuppressive agents after transplantation might worsen malignant tumors [13, 18]. However, in the present study, there were no instances of tumor-like lesions in the small intestine. In any event, the small intestine is regarded as a rare site for malignant tumors; these accounts for only 3–5% of all malignant gastrointestinal tumors [19]. Therefore, screening of tumor-like lesions in the small intestine before LT might not be necessary. However, CE is recognized as a minimally invasive and well-tolerated procedure with a high success rate for complete visual investigation of the whole small intestine. Here, we recorded no severe adverse events during and after the procedure, and we therefore consider that CE can be routinely performed before LT to screen for small intestinal tumors analogous to the employment of colonoscopy for colorectal tumors. We consider that CE is less invasive than conventional endoscopy and hope that whole GI screening before LT can be routinely performed by CE in the near future.

There are some limitations to this study. First, our data were obtained from a single center and the number of patients was relatively small. Currently, most of LT has been performed from living donors in Japan, as cases of cadaver donor transplantation are insufficient to meet demand. Hence, subject accumulation is relatively challenging.

Second, in this study, we could not perform follow-up CE. The most important reason was an ethical problem. In all likelihood, after LT, most portal hypertension issues would improve. Therefore, the Ethics Committee of our hospital refused CE after LT. We predict that changes of endoscopic findings could be detected if follow-up CE was performed.

## 5. Conclusions

In conclusion, we provide evidence that small bowel examination by capsule endoscopy for patients before liver transplantation can be performed safely and is justified because of the detection of a high rate of abnormal lesions specific to PHE, especially in patients with history of EV therapy or bleeding, red color sign, and presence of PHG or HCC.

## Figures and Tables

**Table 1:** Clinical characteristics of patients.

Age (years, mean ± SD)	56.3 ± 8.9
Sex (men/women)	13/18
Etiology	11/6/5/5/4
(HCV/NASH/alcohol/PBC/others)	
HCC (yes/no)	6/25
Portal thrombus (yes/no)	2/29
Child–Pugh score (mean ± SD)	11.0 ± 1.6
MELD score (mean ± SD)	17.0 ± 4.6
Upper and lower endoscopic findings	
E. varices	23 (74%)
History of therapy	13 (42%)
History of bleeding	8 (26%)
G. varices	5 (16%)
PHG	9 (29%)
PHC	9 (29%)

**Table 2:** Comparison of safety of VCE in patients who needed LT and in control patients.

	LT group (*N* = 31)	Control (*N* = 139)	*P* value
Complete small bowel	27/31	128/139	0.67^*∗*^
Examination rate	(87%)	(92%)	
Gastric transit time (min)	34.2 ± 10.9	40.1 ± 5.0	0.62^*∗∗*^
Small intestinal transit time (min)	320.5 ± 24.2	290.5 ± 11.0	0.26^*∗∗*^
Incidence			
Retention	0	0	
Others	1 (dysphagia)	0	

^*∗*^chi-squared test; ^*∗∗*^Student's *t*-test.

**Table 3:** Endoscopic findings in the small bowel.

Finding	*N* (%)
Edema	20 (69%)
Red spots	17 (58%)
Angioectasia	8 (28%)
Erosion	5 (17%)
Varix	2 (7%)
Active bleeding	1 (3%)

**Table 4:** Comparison of the low score and high score patient groups.

	Low score group (*N* = 13)	High score group (*N* = 17)	*P* value
History of EV therapy	2	11	0.008
History of EV bleeding	1	7	0.04
RC sign	0	6	0.006
GV	3	2	0.62
PHG	1	8	0.02
PHC	2	7	0.14
HCC	1	5	0.02
Portal thrombus	2	1	0.76
Child–Pugh score	11.0	10.9	0.89
MELD score	17.2	16.7	0.89

Chi-squared test: history of EV therapy, history of EV bleeding, PHG, and HCC.
