# Corrigendum to “Safety and Efficacy of Small Bowel Examination by Capsule Endoscopy for Patients before Liver Transplantation”

**DOI:** 10.1155/2017/6418529

**Published:** 2017-07-03

**Authors:** Seiji Kawano, Akinobu Takaki, Masaya Iwamuro, Tetsuya Yasunaka, Yoshiyasu Kono, Kou Miura, Toshihiro Inokuchi, Yoshiro Kawahara, Yuzo Umeda, Takahito Yagi, Hiroyuki Okada

**Affiliations:** ^1^Department of Gastroenterology and Hepatology, Okayama University Graduate School of Medicine, Dentistry, and Pharmaceutical Sciences, Okayama, Japan; ^2^Department of Endoscopy, Okayama University Hospital, Okayama, Japan; ^3^Department of Hepato-Biliary-Pancreatic Surgery, Okayama University Hospital, Okayama, Japan

In the article titled “Safety and Efficacy of Small Bowel Examination by Capsule Endoscopy for Patients before Liver Transplantation” [1], the first and last names of all the authors were reversed. The revised authors' list is shown above.
